# Single Plant Derived Nanotechnology for Synergistic Antibacterial Therapies

**DOI:** 10.1371/journal.pone.0163270

**Published:** 2016-09-29

**Authors:** Jhansi R. Kalluri, Roberto Gonzalez-Rodriguez, Phil S. Hartman, Armando Loni, Leigh T. Canham, Jeffery L. Coffer

**Affiliations:** 1 Department of Chemistry, Texas Christian University, Fort Worth, TX, 76129, United States of America; 2 Department of Biology, Texas Christian University, Fort Worth, TX 76129, United States of America; 3 pSiMedica Ltd., Malvern Hills Science Park, Geraldine Road, Malvern, Worcestershire, WR14 3 SZ, United Kingdom; 4 Nanoscale Physics Research Laboratory, School of Physics and Astronomy, University of Birmingham, Edgbaston B152TT, United Kingdom; Helsingin Yliopisto, FINLAND

## Abstract

Multiple new approaches to tackle multidrug resistant infections are urgently needed and under evaluation. One nanotechnology-based approach to delivering new relevant therapeutics involves silicon accumulator plants serving as a viable silicon source in green routes for the fabrication of the nanoscale drug delivery carrier porous silicon (pSi). If the selected plant leaf components contain medicinally-active species as well, then a single substance can provide not only the nanoscale high surface area drug delivery carrier, but the drug itself. With this idea in mind, porous silicon was fabricated from joints of the silicon accumulator plant *Bambuseae* (Tabasheer) and loaded with an antibacterial extract originating from leaves of the same type of plant (*Bambuseae arundinacea*). Preparation of porous silicon from Tabasheer includes extraction of biogenic silica from the ground plant by calcination, followed by reduction with magnesium in the presence of sodium chloride, thereby acting as a thermal moderator that helps to retain the mesoporous structure of the feedstock. The purified product was characterized by a combination of scanning electron microscopy (SEM), energy dispersive X-ray analysis (EDX), X-ray diffraction (XRD), Raman spectroscopy, transmission electron microscopy (TEM), and low temperature nitrogen gas adsorption measurements. Antimicrobial activity and minimum inhibitory concentration of a leaf extract of *Bambuseae arundinacea* was tested against the bacteria *Escherichia Coli* (*E*. *Coli*) and *Staphylococcus aureus* (*S*. *Aureus*), along with the fungus *Candida albicans* (*C*. *Albicans*). A *S*. *aureus* active ethanolic leaf extract was loaded into the above Tabasheer-derived porous silicon. Initial studies indicate sustained *in vitro* antibacterial activity of the extract-loaded plant derived pSi (25 wt %, TGA), as measured by disk diffusion inhibitory zone assays. Subsequent chromatographic separation of this extract revealed that the active antimicrobial species present include stigmasterol and 2,6-dimethoxy-p-benzoquinone.

## Introduction

The rise in drug-resistant pathogens is of major importance and rightly receiving significant worldwide attention [1]. A multitude of alternatives to antibiotics are being explored, including the use of specific metallic ions, biomolecules and even predatory pathogens themselves like phages [2]. There is also increasing interest in adaptable biomimetic strategies to combat multi-drug resistant (MDR) strains of bacteria. Of these, plant-derived approaches are particularly promising for supplying indigenous low-cost therapies [3]. They are also highly amenable to synergistic approaches where a multitude of antimicrobial weapons are employed [4]. Whilst phytochemistry is an established field, and increasing research is now being undertaken on their utility with regard antibiotic-resistant microorganisms [5–7], we are not aware of prior studies combining biodegradable biomaterial and antimicrobial extraction from a single type of plant.

We chose bamboo as our prototype example here, because this plant is highly abundant, rapidly growing, can excrete a form of relatively pure mesoporous silica from its stems [8] and some preliminary studies claim its leaf extracts may exhibit significant activity against MDR bacteria [9–10]. The use of nanostructured mesoporous biomaterials to deliver plant antimicrobial extracts is also very well aligned with synergistic approaches, since these types of biomaterials, such as “black silicon”, have been shown to have mechano-responsive bactericidal activity via their surface topography [11] and can provide loading with antibacterial-active metal ions such as silver [12] and sustained release of antimicrobial peptides & enzymes.

In order to achieve sustained delivery from a biodegradable silicon-based carrier, for this study we selected mesoporous silicon (pSi). Porous silicon, with its nanoscale architecture and tunable surface chemistry, is a promising resorbable nontoxic biomaterial for a broad variety of applications: *in vivo* biosensors [13], and tissue engineering [14], and carrier for controlled drug delivery [15–17]. The most conventional methods for preparing biodegradable mesoporous silicon particles are anodization of single crystal silicon wafers [18] and metal-assisted stain etching processes [19]. Rather than employing these methods—that use corrosive reagents (hydrofluoric acid, HF) and expensive Si feed stocks—we select the eco-friendly alternative of producing pSi from silicon accumulator plants/agriculture waste [20]. Multiple land-based plants typically absorb bio-available silicon in the form of organic silicates such as monosilicic or orthosilicic acid [21]. Among well-known silicon accumulator plant (Tabasheer) have a relatively high percentage of organic silica (95%) [8] compared to other silicon accumulator plants which typically contain about 25–30% silica [22].

Apart from being an excellent source of silica, another important feature of *Bambuseae* is that it has been widely reported that the leaves, root, bark and other constituent parts possess therapeutic activities used to alleviate some medical ailments because of its phytomedical constituents. In particular, pharmacological studies on different parts of *Bambuseae arundinacea* shows that this plant possesses a variety of biological activities including antibacterial activity [23–25]. It should also be emphasized that the Tabasheer component of *Bambuseae* alone has an extensive history of medicinal use, primarily in herbal treatments as an antipyretic, antispasmodic, and antiparalytic [26].

Thus using Bamboo as a source of silica as well as ideally a variety of bioactive components, our aim is to develop an eco-friendly drug delivery matrix where the carrier (pSi) is derived from the stem and drug (antibacterial agents in this case) is gleaned from the leaf of the same plant. This goal is accomplished by fabricating pSi from Tabasheer powder obtained from the nodal joints of the bamboo tree and loaded with an antibacterial extract originating from leaves of the same type of plant. The fabrication process, extraction of antibacterial agents from the leaf and subsequent loading of antibacterial extracts into plant-derived pSi, evaluation of sustained release of antibacterials from the drug carrier, and identification process of some of the compounds responsible for antibacterial activity are discussed herein.

## Materials and Methods

### Fabrication of Plant derived Silicon

In a typical experiment 0.5 g of Tabasheer powder (ground from commercially-available pieces (Bristol Botanicals Ltd.) using a ball mill) was suspended in 20 mL 10% hydrochloric acid. The solution was stirred constantly at 100°C for 2 hrs by replenishing hydrochloric acid for every 30 min. After 2 hrs of heating the powder was washed with D.I water, filtered, and vacuum dried overnight. The dry HCl washed powder was loaded in a quartz boat (1.5 x10 cm) and calcinated at 500°C in presence of O_2_ for 2 hrs in a tube furnace. The calcinated powder was then added to magnesium powder in the molar ratio of 1:2 (SiO_2_: Mg). Sodium chloride was added to the above reaction blend in the ratio of 1:1 by weight. Then this mixture was reduced at 600°C for 2 hrs in presence of Argon in a tube furnace. After reduction the residual magnesium phases were removed by heating the reduced sample with 36% HCl at 70°C for 1 hr.

### Extraction of antibacterials from *Bambuseae arundinacea* leaves

Fresh leaves of the plant *Bambuseae arundinacea* were collected in June 2013 from Eluru, Andhra Pradesh, India, under the supervision of one of the authors (JK) on the private property of Mr. Veerakumar Chintapalli, with his permission. This is a plant common to the region that does not fall into a category of endangered or protected species (according to the International Union for the Conservation of Nature (IUNC). These leaves were subsequently examined by Barney L. Lipscomb, systematic botanist from Botanical Research Institute of Texas, Fort Worth, TX, USA. The plant leaves were shade dried for two days and coarsely powdered (10 g) and extracted with ethanol (100 mL) at a temperature of 85°C in a Soxlet extractor. After 3 hrs of extraction, extract was concentrated through use of a rotary vacuum evaporator and the dry extract (0.5 g) was stored at -4°C.

### Loading of ethanolic leaf extract into tabasheer derived pSi

Small amounts of pSi derived from Tabasheer (typically ~10 mg), fabricated according to the above process, were soaked in 0.120 g of extract dissolved in 1 mL of ethanol for 2 hrs. Then this mixture was heated at 70°C for 30 min. The above mixture was centrifuged; supernatant was removed, and washed with sterile water twice and dried under vacuum overnight.

### Disc diffusion assay

Antibacterial activity of the extract and extract loaded in pSi was tested using a disc diffusion assay. Dry plant extract (1.3 mg) was dissolved in 10% DMSO and 60 μL of extract was placed aseptically on a sterile filter paper disc (diameter 6 mm) and transferred to an inoculated Luria-Bertani (LB) agar plate seeded with the bacterial culture (10^7^ CFU/ml, 24 hrs). Discs injected with 60 μL of 10% DMSO were used as a negative control. Then the plates were incubated at 37°C for 18 hrs. After the allotted period, the inhibition zones formed were measured. All the samples were run in triplicate. pSi powder (20 mg) loaded with ethanol leaf extract was tested for the evaluation of sustained antibacterial activity of the leaf extract loaded pSi. pSi powder (20 mg) was suspended in 0.6 mL of sterile DI water in a 10 mL vial. The vial was continuously rotated using an agitating apparatus at 37°C. After 24 hrs of agitation the vial was centrifuged and the supernatant was drawn carefully into a sterile vial. This procedure was repeated after replenishing 0.6 mL sterile DI water into the vial. Discs injected with 60 μL of sterile DI water were used as a negative control. A 60 μL fraction of the supernatant was injected onto paper discs (triplicate) and transferred to an agar plate seeded with bacterial culture (10^7^ CFU/ml, 24 hrs) and incubated at 37°C for 18 hrs to measure inhibition zones.

### Micro broth dilution assay

An ethanol leaf extract (5.2 mg/mL), derived as described above, and was dissolved in dissolved in 10% DMSO. Dilutions of crude extract were prepared in Luria-Bertani (LB) broth by adding 50 μL of Luria-Bertani (LB) broth to 50 μL of extract. The extract solutions were then serially diluted (1:1) down to give 2.6, 1.3, 0.65, 0.325, 0.1625 mg/mL concentrations of crude extract. Bacteria at a concentration of 10^7^ CFU/ml were added and after 18 hrs of incubation the optical densities of the samples were measured at 600 nm. Sterile water added to Luria-Bertani (LB) broth was used as a negative control. For this extract, a MIC value of 1.3 mg/mL was obtained.

## Results and Discussion

**Fig 1:**
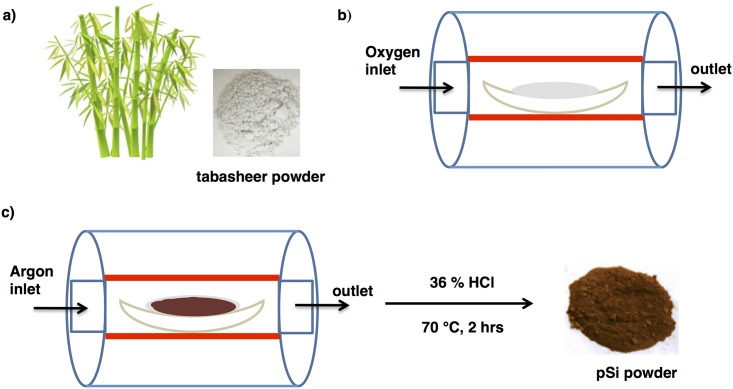
Eco-friendly fabrication route for extraction of pSi from tabasheer. Optical image of grounded tabasheer powder obtained from Bamboo, **b**) calcination of 10% HCl washed tabasheer in a tube furnace, **c**) magnesiothermic reaction of calcinated tabasheer powder, and pure pSi powder obtained after washing with 36% HCl.

**Fig 2:**
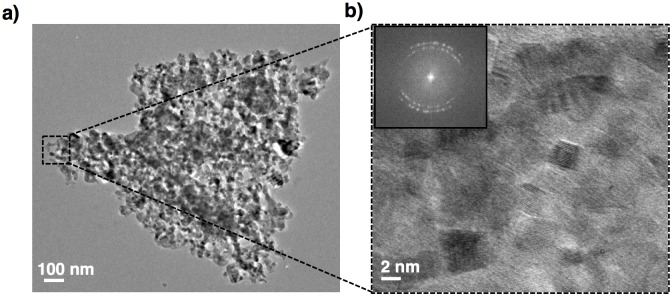
TEM images of plant derived pSi. **a**) low magnification image of free standing pSi microparticle, **b**) high resolution TEM image with a small silicon crystalline domains in the range of 4–8 nm, inset shows polycrystalline orientation of silicon nanocrystallites (scale bar of **a**) = 100 nm; **b**) = 2 nm).

The individual steps of fabrication route are summarized in Fig 1. Commercially-available pieces of Tabasheer (*Concretio silicea bambuseae* from Bristol Botanicals Ltd.) were first ground to a fine powder using a commercial ball mill. An initial washing process of the Tabasheer powder with dilute hydrochloric acid (10%) allowed the removal of metallic residues possibly deposited during the plant grinding process. This dried powder was then calcinated in the presence of oxygen to extract biogenic silica from the purified Tabasheer powder. The extracted silica was subsequently converted to pSi by magnesiothermic reduction using magnesium powder in a molar ratio of 1:2 (SiO_2_: Mg). [20] The highly exothermic nature of this reaction between silica and magnesium was controlled by adding sodium chloride as a thermal moderator, as well as to maintain porosity. Washing the product of this reaction with 36% hydrochloric acid dissolves all the traces of magnesium which was confirmed by energy dispersive X-ray analysis (EDX) (S1 Fig). This magnesiothermic reduction resulted in the formation of pSi microparticles comprised of an aggregate of small nanoparticles creating nanopores along the microparticle surface, as gauged by transmission electron microscopy (Fig 2a). High resolution TEM analysis (Fig 2b) reveals the presence of numerous small Si nanocrystals embedded in an amorphous matrix; lattice imaging provides evidence for the cubic phase structure of these nanocrystals, with the <111> lattice spacing of 0.310 nm readily visualized. Electron diffraction patterns (inset Fig 2b) further support the characterization of the pSi derived from the magnesium-reduced calcinated Tabasheer as polycrystalline in nature.

**Fig 3:**
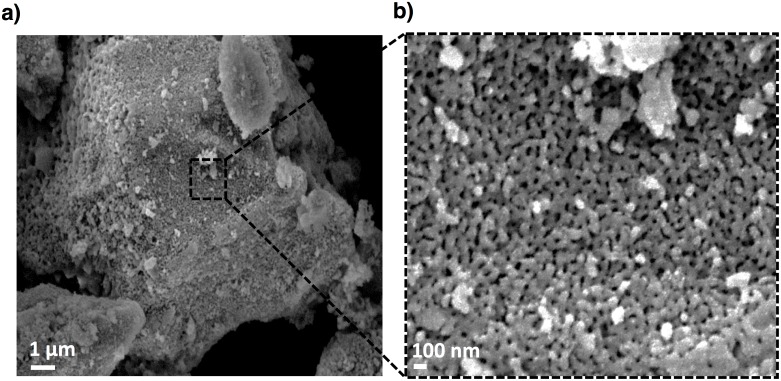
FESEM images and observed pSi microparticle surface morphology. (**a**) free standing pSi microparticle from magnesium reduced tabasheer, reaction final product, (**b**) individual nanopores along the pSi particle surface (scale bar of (**a**) = 1 μm, (**b**) = 100 nm.

In a manner complementary to TEM, analysis of a typical reaction product by field emission scanning electron microscopy (FESEM) reveals a microparticle morphology with a wide distribution of sizes (mean diameter of 24±20 μm) and uniformly distributed pores with a mean size of 32 nm (± 8 nm) on the silicon microparticle surface (Fig 3b). Consistent with FFT diffraction analysis, XRD patterns associated with this type of magnesium-reduced calcinated Tabasheer sample shows peaks at 2θ = 28°, 47°, and 56°, ascribed to crystalline silicon reflections associated with the (111), (220), and (311) indices, respectively (S2 Fig). Raman spectroscopic measurements indicate the characteristic crystalline Si phonon at 521 cm^-1^ (S3 Fig). The presence of a *v*(Si-O-Si) stretching vibration at 1087 cm^-1^ (FT IR, S4 Fig) is consistent with significant oxide content in the matrix.

This pSi produced by the magnesium-reduction of calcinated Tabasheer has a surface area of 178 m^2^/g, a pore volume of 0.506 ml/g, and an average pore diameter of 11 nm according to the nitrogen gas adsorption data (S5 Fig). Such values were derived from nitrogen gas adsorption/desorption data, with computational analysis of the isotherms based on the BET (Brunauer-Emmett-Teller) and / or BJH (Barret-Joyner-Halenda) methods, underpinned by the classical Kelvin equation, which facilitated the calculation of surface area, pore volume (from the adsorption isotherm), average pore size and pore size distribution.

The phytochemicals associated with possible antibacterial activity were extracted from dried leaves of the plant *Bambuseae arundinacea*. Shade-dried powdered leaves were subjected to a continuous hot extraction with ethanol (95%) in a Soxhlet extractor, followed by concentrating the extract under vacuum. The dried extract dissolved in dimethyl sulphoxide (10%) showed activity (evaluated by disc diffusion and microbroth dilution assays) against gram positive bacteria *Staphylococcus aureus* (ATCC 25923) in contrast to the two other tested species—gram negative *Escherichia coli* (ATCC 25922), and fungal strain *Candida albicans* (ATCC 10231); this extract showed no measureable activity versus the latter two species. The detailed process of extraction and antibacterial assays are described in the methods section.

**Fig 4:**
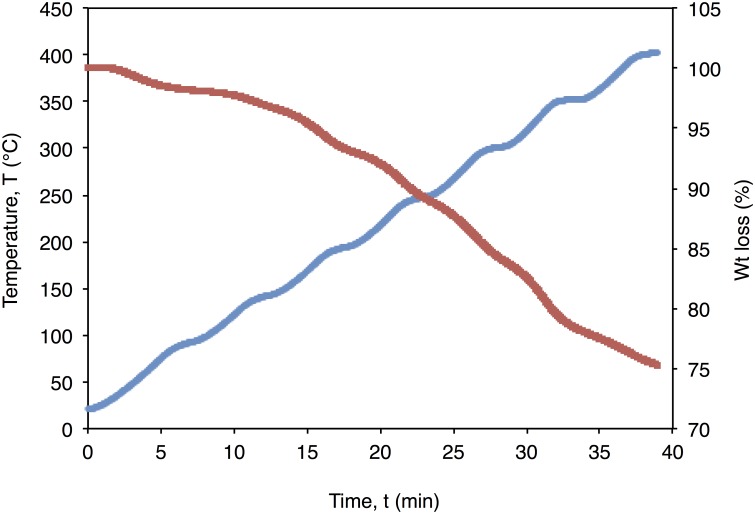
Thermo gravimetric analysis (TGA) of the extract loaded into pSi. Weight loss curve (in red), heating curve (in blue) of crude leaf extract loaded pSi (10 mg) was hated in N_2_ at a ramp rate of 10°C/ min to a temperature of 400°C beyond this no further degradation was observed.

**Fig 5:**
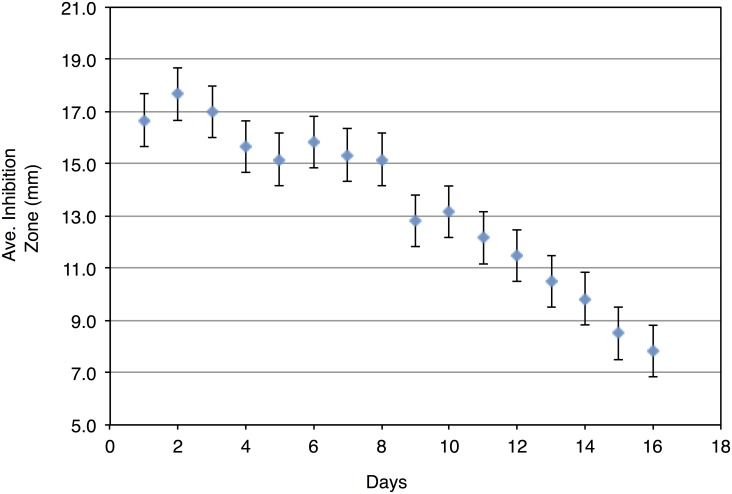
Antibacterial activity of the extract loaded Tabasheer-derived pSi carrier against *S*. *aureus*. Using a disk diffusion assay, the release of antibacterials and associated activity was monitored until the activity ceased.

The leaf derived antibacterial extract was then loaded into the pSi by a wet loading method to give a 25% loading capacity (by weight) as analyzed by thermo-gravimetric analysis (TGA) (Fig 4). Exposure of the extract and the extract-loaded into the plant-derived pSi showed measurable antibacterial activity versus *S*. *aureus*. The measured inhibition zones of the extract and extract-loaded pSi (in dry powder form) are listed in Table 1. Comparable levels of antibacterial activity are noted. The sustained release of active species loaded into the pSi were also monitored for a period of time until any measureable antibacterial activity ceased against *S*. *aureus* using agar plate diffusion assay (Fig 5). Significant antibacterial activity was observed for a period up to 16 days. It should be also emphasized that no measureable antibacterial activity versus *S*. *aureus* has been observed for this pSi component alone (as a control).

**Table 1:** Antibacterial activity (against *S*. *aureus)* of bamboo extract and extract-loaded pSi after 24 h incubation period (at 37°C).

*Material Tested*	*Inhibition Zone (mm)*
Bamboo leaf extract (78 mg)	18
Extract loaded Tabasheer-derived pSi (2.5 mg of pSI with 25% loading)	13

To begin the separation and identification process of the active phytochemicals responsible, the dried leaf extract obtained from *Bambuseae arundinacea* was initially fractionated with four following solvents based on their polarity ranging from low to high: petroleum ether (3x10 mL), chloroform (CHCl_3_, 3x10 mL), ethyl acetate (EtOAc, 3x10 mL) and methanol (MeOH, 3x10 mL). The fractions of each solvent were then combined and dried under vacuum. Each dried fraction was tested for antibacterial activity by plate diffusion assay. Among the four solvent fractions, the petroleum ether CHCl_3_, and EtOAc fractions showed activity against *S*. *aureus* (S1 Table). The highly and equally active CHCl_3_ and EtOAc fractions were then further analyzed to find out the antibacterial agents present. All the four fractions were monitored with thin layer chromatography (TLC) and similar fractions with the same (R_f_ and colour) were combined. The dried combined fractions of CHCl_3_ and EtOAc were subjected to preparative TLC using petroleum ether and chloroform (2:3) as a solvent system, which yielded in five sub fractions. Among these five sub fractions, two sub fractions fraction 5, fraction 8, were active against *S*. *aureus* with inhibition zones of 21 mm, and 22 mm and fraction 9 with an inhibition zone of 11 mm. Fraction 5 and fraction 8 were further purified by preparative TLC using the solvent systems chloroform: ethyl acetate: acetic acid (4.5:4.5:1) and hexane: ethyl acetate (3:2) respectively. The elution of sub fraction 5 yielded fraction 10 and pure compound **1** and sub fraction 8 to afford fraction 11 and compound **2**. Compound **1** was further purified by washing with dichloromethane and methanol. The isolation process, along with the amounts of fractions obtained after each purification step, is listed in supplementary information (S6 and S7 Figs). The compound **1** (yellow powder, ~1 mg) was identified as 2,6-dimethoxycyclohexa-2,5-diene-1,4-dione (2,6 dimethoxy-p-benzoquinone) based on a straightforward assignment of combined spectral data (^1^H NMR, ^13^C NMR, and ESI-MS) and compound **2** (white powder, ~2 mg) identified as 3S,8S,9S,10R,13R,14S,17R)-17-[(E,2R,5S)-5-ethyl-6 methylhept-3-en-2-yl]-10,13-dimethyl-2,3,4,7,8,9,11,12,14,15,16,17 dodecahydro-1H-cyclopentaphenanthren-3-ol (stigmasterol) by comparing its combined spectral data (^1^H NMR, ^13^C NMR, and ESI-MS) to that reported in the literature [27]. Compound **1**, and compound **2** show activity against *S*. *aureus* with individual minimum inhibitory concentrations (MIC) of 35 μg/mL, and 30 μg/mL respectively. The recorded ^1^H NMR, ^13^C NMR, and HRMS spectra for isolated 2, 6 dimethoxy- p-benzoquinone (S8, S9 and S10 Figs), and stigmasterol (S11, S12 and S13 Figs) are listed in supporting information. Both pure compounds have demonstrated antibacterial activity previously [27–29].

The eco-friendly fabrication route described here to extract silicon from Tabasheer resulted in high yields of nanocrystalline silicon/silicon oxide (~70%) with more than ~99% purity (by EDX). This fabrication method is relatively economical and eco-friendly, requiring no specialized equipment or reagents. Use of a thermal moderator (NaCl) also serving as templating agent permits retention of nanoscale features (pore morphology) of the pSi during reduction with magnesium at the modest temperature of 600°C. The pSi formed during this process is highly crystalline, as strongly confirmed by HRTEM lattice imaging, x-ray diffraction (XRD), and Raman spectroscopy. Taken in concert, and recognizing that both FT IR and EDX analyses show significant oxygen content in the matrix, one can view the porous microstructure of this material as series of Si nanocrystals embedded in an amorphous oxide phase. In terms of porosity, analysis of low temperature nitrogen adsorption data in a BET isotherm-type model reveal pore size values in the mesoporous range, with associated surface areas also consistent with this classification.

Antibacterial assays on the leaf extract obtained from *Bambuseae arundinacea* show promising activity against *S*. *aureus*. Incorporation of the antibacterial leaf extract into the pSi rendered us to study the controlled release of the antibacterial agents from the drug carrier matrix. The wet loading method used to load the extract into pSi allows slow diffusion of the phytochemicals into the pores to yield a 25 wt% loading. TGA analysis shows an initial weight loss (<5%) from ambient temperatures to 100°C that is likely due to the volatile components and desorption of physically adsorbed water, with the weight loss from 150 to 400°C assumed to be associated with degradation of bioactive phytochemicals identified during the extraction/separation process.

A steady plateau of inhibition zones (measured at 24h intervals) over the initial 7d window, followed by a gradual diminution in antibacterial activity over the next 9 days, reflects a sustained release of active species *in vitro*. While the duration of the release window for the active therapeutics in this case does not currently extend to the maximum length of that of selected known antibacterial drug delivery systems based on anodized pSi, [30] it does nevertheless demonstrate utility for this system in a possible clinical context. The key fact that relatively straightforward experimental conditions- available globally—are needed to simultaneously provide both drug and nanoscale drug carrier is an encouraging point to stimulate further practical development.

## Conclusions

In summary, this work clearly demonstrates that a single plant type can yield useful therapeutics that are delivered from a drug delivery carrier derived from a silicon accumulator plant. This eco-friendly fabrication route is an ideal alternative to conventional etching methods for preparing oxidized pSi powders of high purity in competitive yields, possessing high surface areas as well. The above approach thereby provides multiple appealing advantages on different levels—with a primary focus on the economy and simplicity in fabrication of nanoscale carriers capable of sustained release, along with incorporation of concomitantly-present active therapeutics. Future work will focus on (i) scalability of the above process, (ii) modification of the pSi scaffold for tunability of degradation to affect drug release, and (iii) an expansion of the range of active therapeutics for incorporation and release from this type of plant-derived nanostructured porous carrier (including other related natural product-derived species). It is perceived that the dual advantage of the single plant source for creating this type of drug delivery carrier matrix would be an ideal low-cost option for countries with extensive resources of silicon accumulator plants in future synergistic drug delivery applications.

## Supporting Information

S1 Fig SEM-EDX analysis on magnesium-reduced Tabasheer. (PDF)

S2 Fig XRD pattern of tabasheer derived pSi. (PDF)

S3 Fig Raman spectrum of porous silicon derived from Tabasheer. (PDF)

S4 Fig FT IR spectrum of porous silicon derived from Tabasheer. (PDF)

S5 Fig Brunauer—Emmett—Teller (BET) analysis on pSi derived from Tabasheer. (PDF)

S6 Fig Flow chart of initial fractionation of *Babusae arundinacea* leaf extract. (PDF)

S7 Fig Flow chart of separation of phytochemicals responsible for antibacterial activity from combined fractions of chloroform and ethyl acetate. Associated characterization data for compounds isolated from these fractions.
(PDF)

S8 Fig ^1^H NMR spectrum of compound 1. (PDF)

S9 Fig ^13^C NMR spectrum of compound 1. (PDF)

S10 Fig HRMS (ESI-TOF) data for compound 1. (PDF)

S11 Fig ^1^H NMR spectrum of compound 2. (PDF)

S12 Fig ^13^C NMR spectrum of compound 2. (PDF)

S13 Fig HRMS (ESI-TOF) data for compound 2. (PDF)

S1 Table Antibacterial activity of four fractions tested against *S*. *aureus* by plate diffusion assay. (PDF)
