# Supplementary material for: Single Plant Derived Nanotechnology for Synergistic Antibacterial Therapies
Source: PLoS One. 2016 Sep 29;11(9):e0163270. doi: 10.1371/journal.pone.0163270 (PMC5042556; doi:10.1371/journal.pone.0163270)
Supplement: S1 Fig — (PDF) [file pone.0163270.s001.pdf]

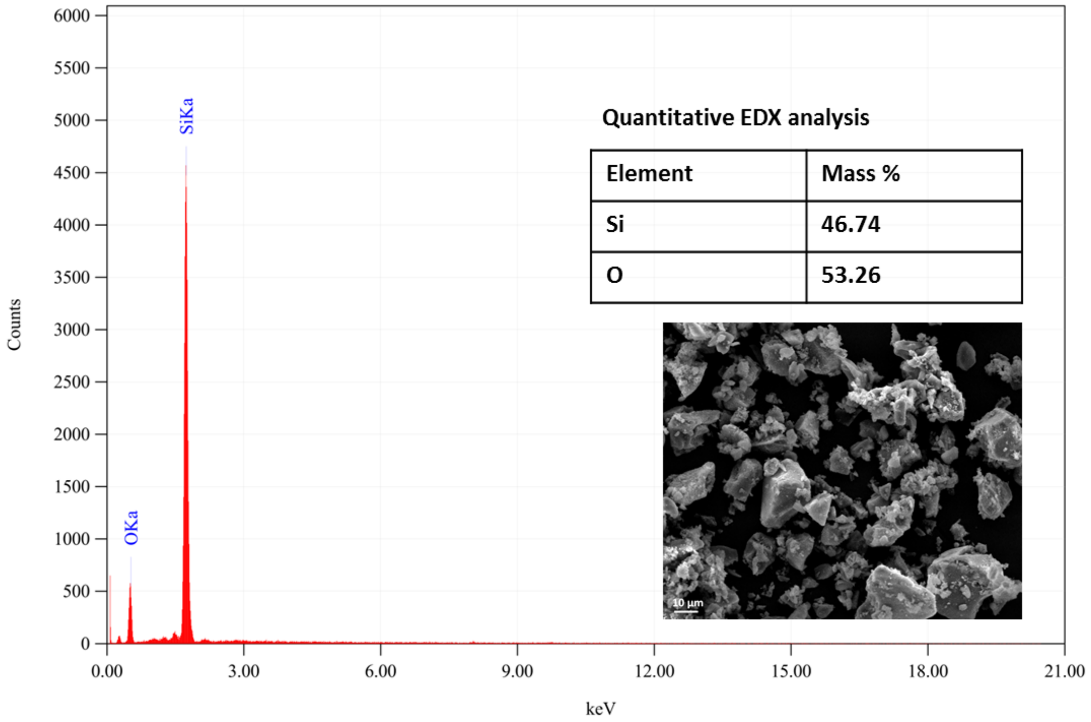

S1 Figure: SEM-EDX analysis on magnesium reduced Tabasheer. Inset shows an SEM image of the pSi produced by a magnesiothermic reduction reaction after leaching with 36% hydrochloric acid (scale bar – 10 µm).

Scanning Electron Microscopy (SEM): Particle size distribution and elemental analysis were performed on all porous silicon samples using a field emission scanning electron microscope (FE-SEM), JEOL 7100. Samples were mounted on a metal (Al) stub with the aid of adhesive carbon tape, and coated with 5 nm thickness of gold (when necessary to eliminate charging).

Transmission Electron Microscopy (TEM): TEM analysis was conducted with a JEOL TEM 2100 operating at 200 kV. Samples were prepared by adding 0.5 mg of a given porous silicon powder to 1 mL of acetone, sonicated for 2 min, with drops placed on a copper grid and allowed to dry.

Thermogravimetric analysis (TGA): TGA was performed by using a Seiko SII model *SSC/5200 TGA* at a heating rate of 10°C/min up to 400°C under nitrogen.
