# Supplementary material for: Single Plant Derived Nanotechnology for Synergistic Antibacterial Therapies
Source: PLoS One. 2016 Sep 29;11(9):e0163270. doi: 10.1371/journal.pone.0163270 (PMC5042556; doi:10.1371/journal.pone.0163270)
Supplement: S2 Fig — (PDF) [file pone.0163270.s002.pdf]

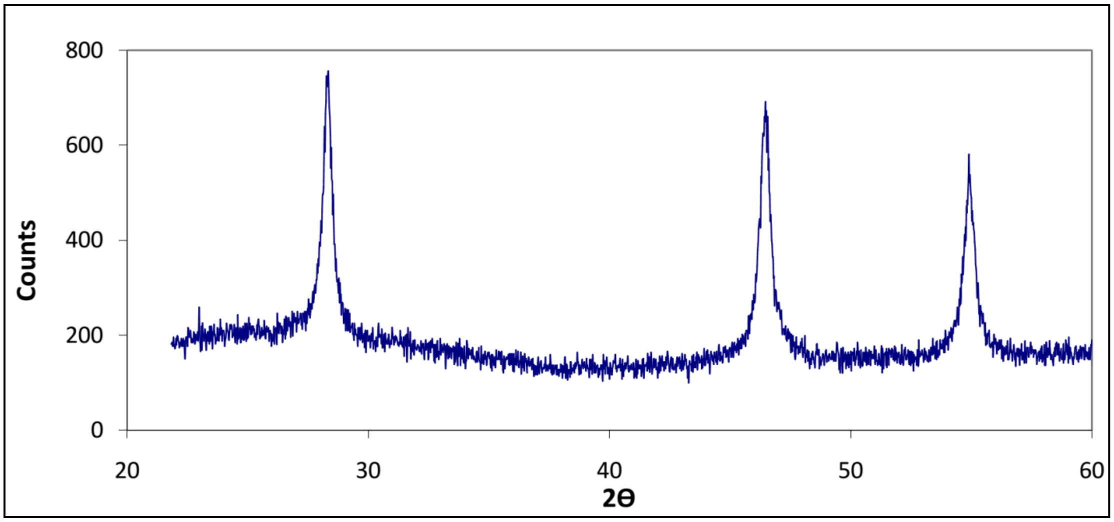

S2 Figure: XRD pattern of Tabasheer derived pSi. Peaks at 2θ = 28°, 47°, and 56° are ascribed to crystalline silicon reflections of (111), (220), and (311).

X-ray diffraction (XRD): XRD patterns were carried out using a Phillips 3100 X-ray Powder diffractometer with Cu Kα radiation operating at 35 kV.
