# Supplementary material for: Single Plant Derived Nanotechnology for Synergistic Antibacterial Therapies
Source: PLoS One. 2016 Sep 29;11(9):e0163270. doi: 10.1371/journal.pone.0163270 (PMC5042556; doi:10.1371/journal.pone.0163270)
Supplement: S3 Fig — (PDF) [file pone.0163270.s003.pdf]

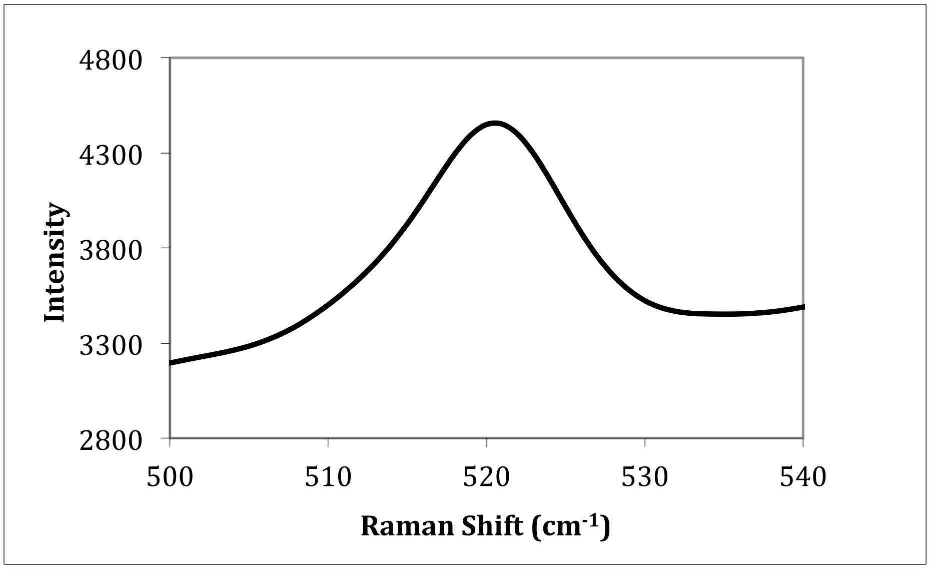

S3 Figure: Raman spectrum of porous silicon derived from Tabasheer.

Raman: Raman spectrometric analysis was performed using a DeltaNu System with a 785 nm laser source.
