# Supplementary material for: Single Plant Derived Nanotechnology for Synergistic Antibacterial Therapies
Source: PLoS One. 2016 Sep 29;11(9):e0163270. doi: 10.1371/journal.pone.0163270 (PMC5042556; doi:10.1371/journal.pone.0163270)
Supplement: S4 Fig — (PDF) [file pone.0163270.s004.pdf]

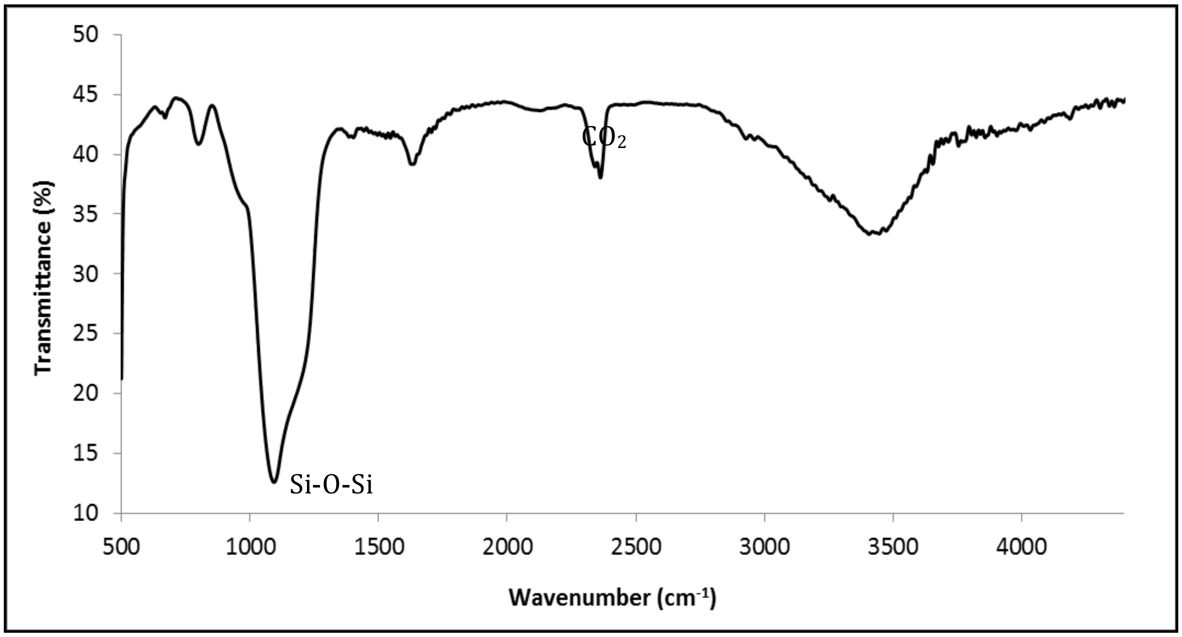

S4 Figure: FTIR spectrum of porous silicon derived from Tabasheer.

FT IR: Samples were prepared in the form of KBr pellets (1% w/w sample concentration) and analyzed using a MIDAC M4000 series spectrometer.
