# Supplementary material for: Single Plant Derived Nanotechnology for Synergistic Antibacterial Therapies
Source: PLoS One. 2016 Sep 29;11(9):e0163270. doi: 10.1371/journal.pone.0163270 (PMC5042556; doi:10.1371/journal.pone.0163270)
Supplement: S5 Fig — (PDF) [file pone.0163270.s005.pdf]

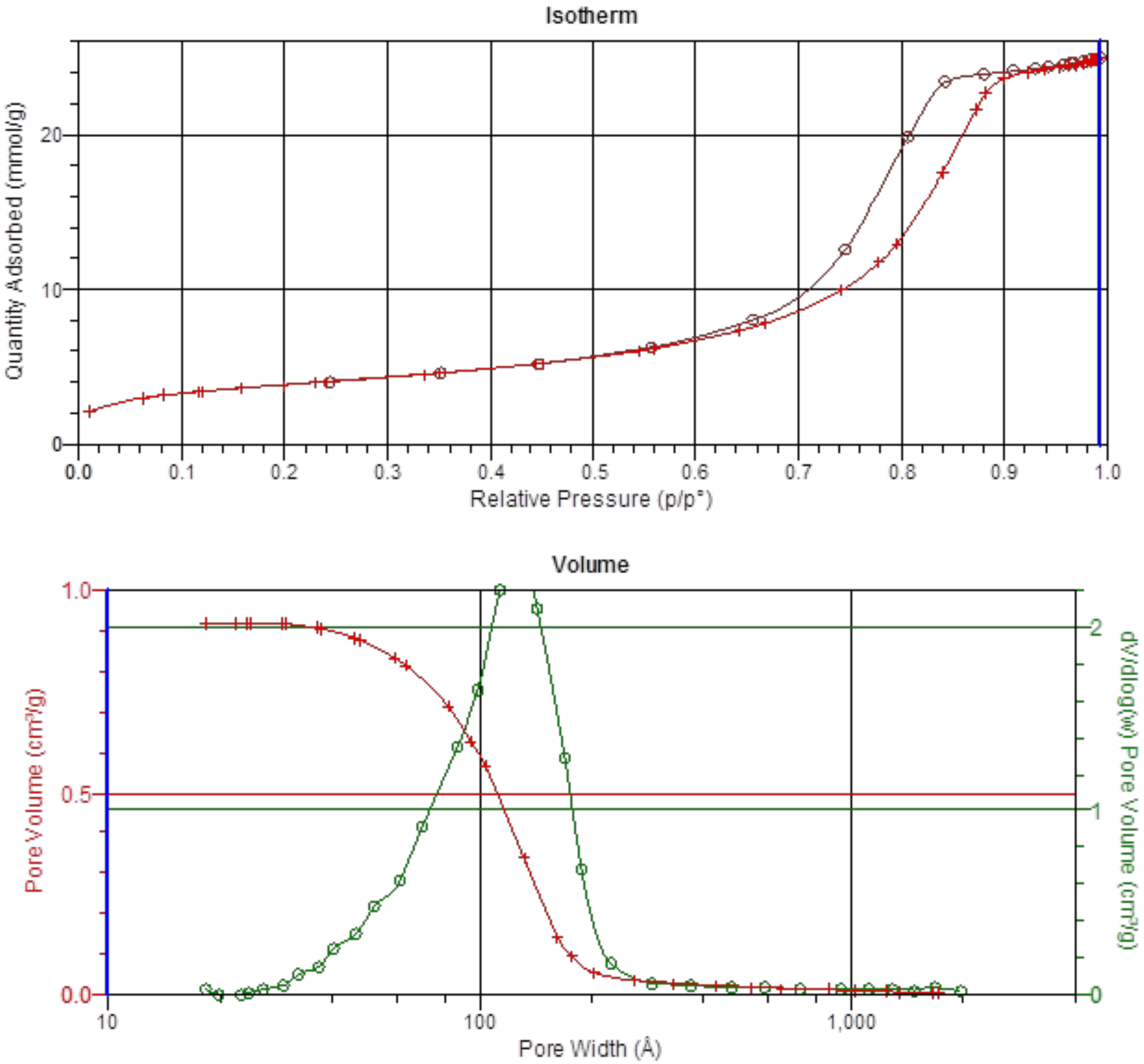

S5 Figure: Nitrogen adsorption isotherms and associated Brunauer–Emmett–Teller (BET) analysis of Tabasheer material.

Isotherm measurements: Nitrogen gas adsorption/desorption was carried out using a Micromeritics Tristar 3000 instrument.
