# Supplementary material for: Single Plant Derived Nanotechnology for Synergistic Antibacterial Therapies
Source: PLoS One. 2016 Sep 29;11(9):e0163270. doi: 10.1371/journal.pone.0163270 (PMC5042556; doi:10.1371/journal.pone.0163270)
Supplement: S6 Fig — (PDF) [file pone.0163270.s006.pdf]

S1 Table: Antibacterial activity of four fractions tested against *S. aureus* (triplicate) by plate diffusion assay

Fraction No.	1	2	3	4
Inhibition Zone (mm)	9 ± 2	16 ± 2	17 ± 1	-
