# Supplementary material for: Single Plant Derived Nanotechnology for Synergistic Antibacterial Therapies
Source: PLoS One. 2016 Sep 29;11(9):e0163270. doi: 10.1371/journal.pone.0163270 (PMC5042556; doi:10.1371/journal.pone.0163270)
Supplement: S7 Fig — Associated characterization data for compounds isolated from these fractions. (PDF) [file pone.0163270.s007.pdf]

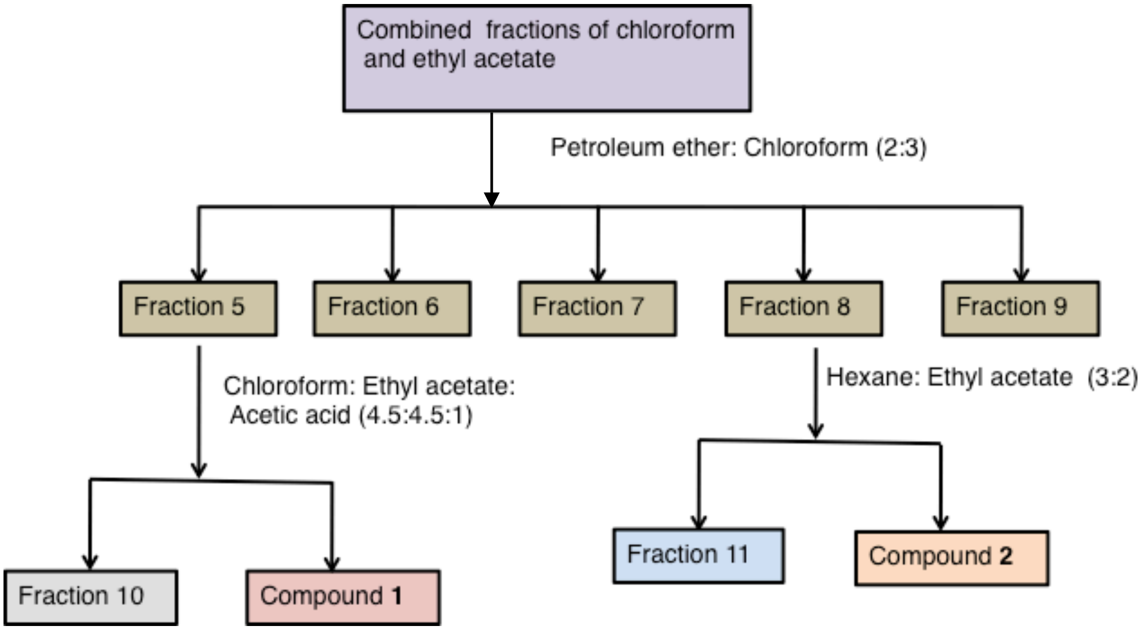

S7 Figure: Flow chart diagram of separation of photochemicals responsible for antibacterial activity from combined fractions of chloroform and ethyl acetate**.** After initial fractionation, combined active fractions of chloroform and ethyl acetate were further purified by preparative TLC to give five sub fractions. Active sub fractions were purified by TLC to afford pure compounds **1**&**2**.

Characterization Data for Compounds 1 and 2

Compound **1**: TLC (Chloroform: Ethyl acetate: Acetic acid), 45:45:10 v/v): $R_f = 0.7$; ^{1}H NMR (400 MHz, $CDCl_3$): δ 5.88 (s, 1H), 3.84 (s, 3H); ^{13}C NMR (400 MHz, $CDCl_3$): δ 186.84, 176.67, 157.33, 107.42, and 56.48; HRMS (ESI-TOF) m/z calcd. for $C_8H_8O_4$, 168.0823; found 169.1132 (M+H).
Compound **2**: TLC (Hexane : Ethyl acetate , 60:40 v/v): $R_f = 0.3$; ^{1}H NMR (400 MHz, $CDCl_3$): δ 5.38 (d, 1H, J = 5.2 Hz), 5.16 (dd, 1H, *J* = 8.8, 8.8 Hz), 5.06 (dd, 1H, *J* = 8.8, 8.8 Hz), 3.54 (m, 1H), 2.29 (m, 2H), 2.03 (m, 3H), 1.85 (m, 2H), 1.72-1.41 (m, 10H), 1.27-1.18 (m, 14H), 1.15-1.03 (m, 6H), 0.90-0.82 (m, 13 H), 0.72 (s, 2H); ^{13}C NMR (400 MHz, CDCl3): δ 140.76, 138.32, 129.29, 122.71, 71.81, 56.88, 55.97, 51.25, 50.17, 42.30, 42.22, 40.50, 39.69, 37.27, 36.52, 31.91, 31.89, 31.66, 28.93, 25.42, 24.37, 21.23, 21.09, 19.41, 18.99, 12.26, 12.06; HRMS (ESI-TOF) m/z calcd. for $C_{29}H_{48}O$, 412.3705; found 395.4788 (M-17), corresponds to loss of hydroxyl group.

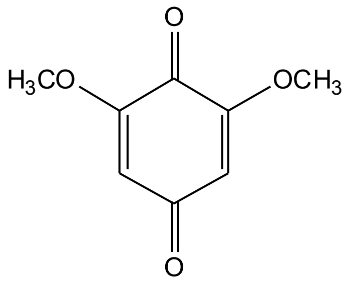

Compound **1**

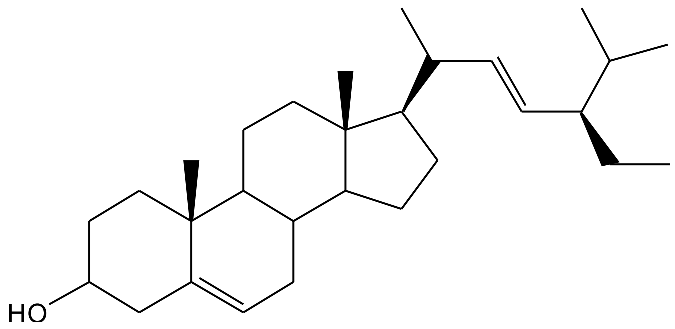

Compound **2**

Mass Spectrometry (ESI-MS): ESI mass spectroscopy analysis was done using an Agilent Technologies 6224 TOF MS system.

NMR: ^{1}H, ^{13}C NMR spectra were recorded on selected extract samples using a Bruker Asend 400
