# Supplementary figures and images for: Single Plant Derived Nanotechnology for Synergistic Antibacterial Therapies
Source: PLoS One. 2016 Sep 29;11(9):e0163270. doi: 10.1371/journal.pone.0163270 (PMC5042556; doi:10.1371/journal.pone.0163270)

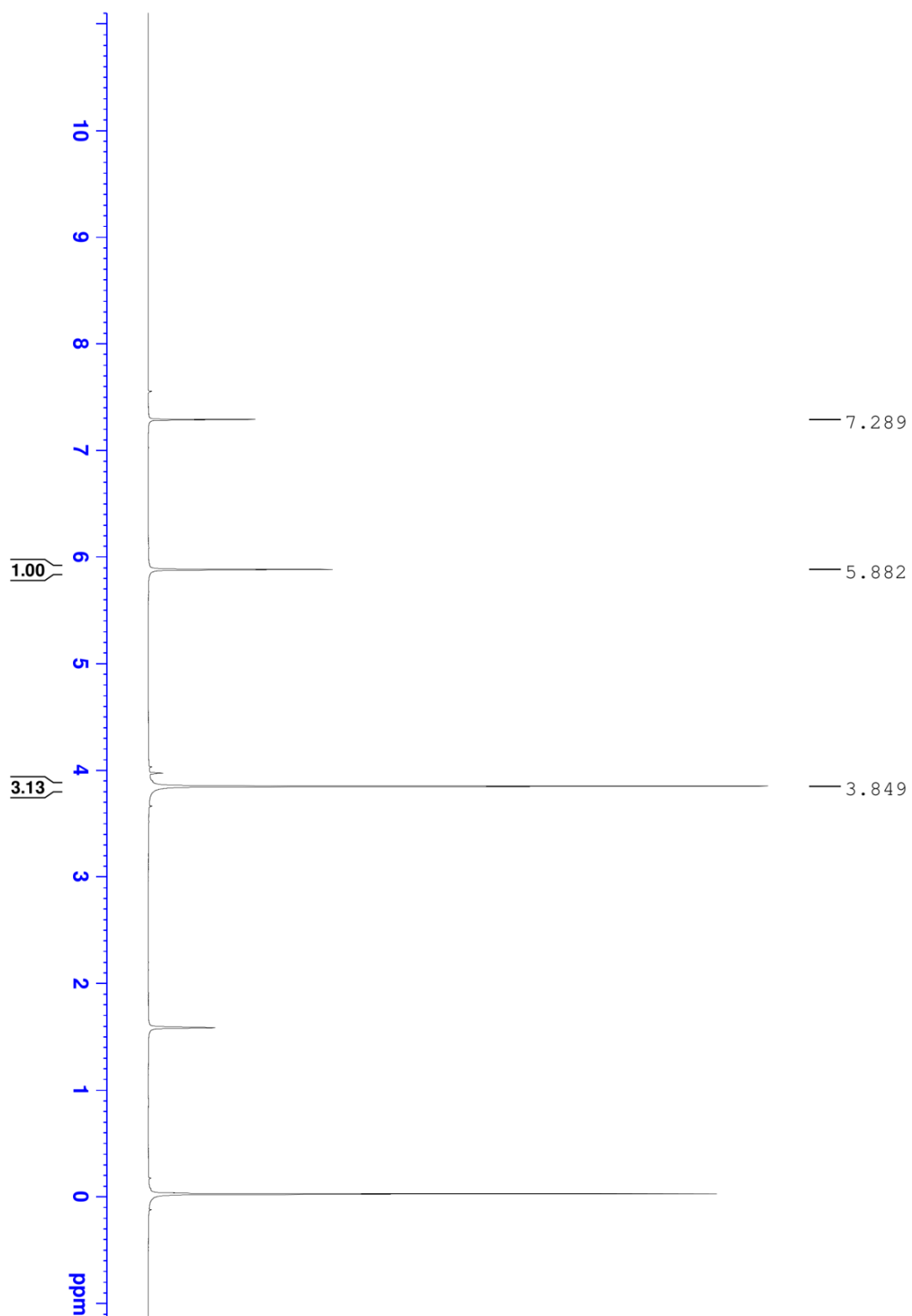

**S8 Figure:** <sup>1</sup>H NMR spectrum of compound (1) in CDCl<sub>3</sub> (400 MHz).

Supplement: S8 Fig — (PDF) [file pone.0163270.s008.pdf]

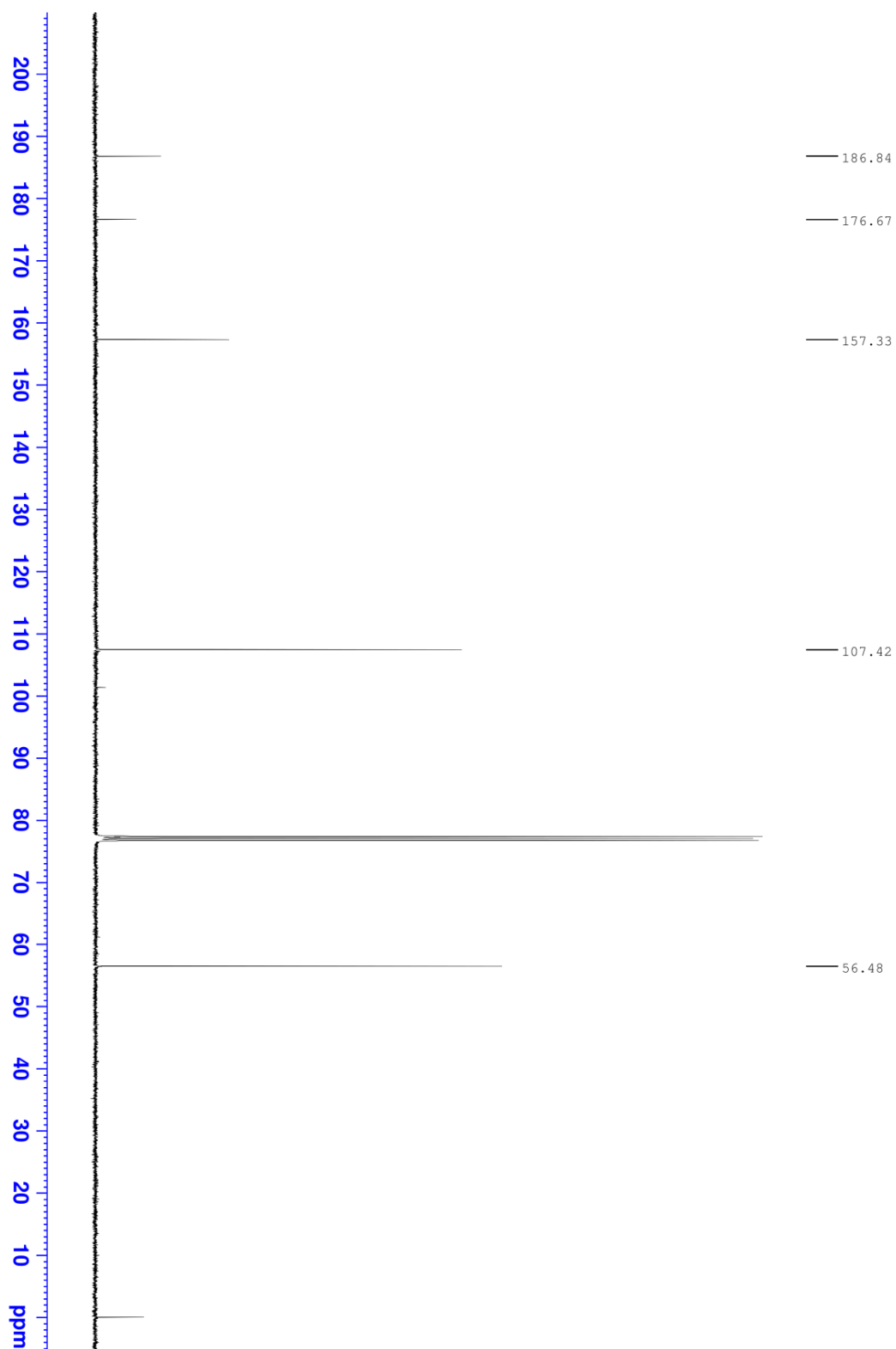

**S9 Figure:**  $^{13}\text{C}$  NMR spectrum of compound **1** in  $\text{CDCl}_3$  (400 MHz).

Supplement: S9 Fig — (PDF) [file pone.0163270.s009.pdf]

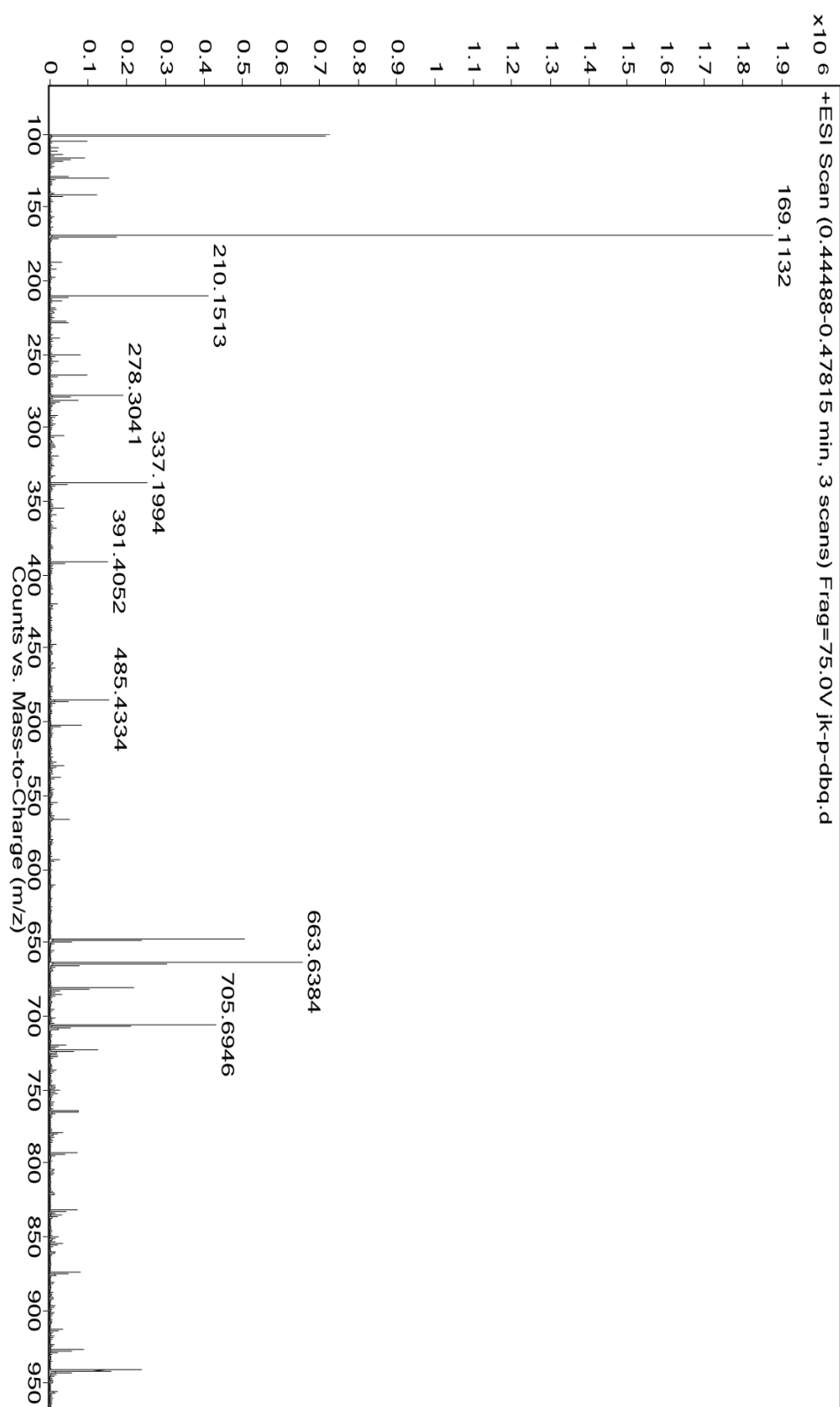

**S10 Figure:** HRMS (ESI-TOF) data for compound **1**.

Supplement: S10 Fig — (PDF) [file pone.0163270.s010.pdf]

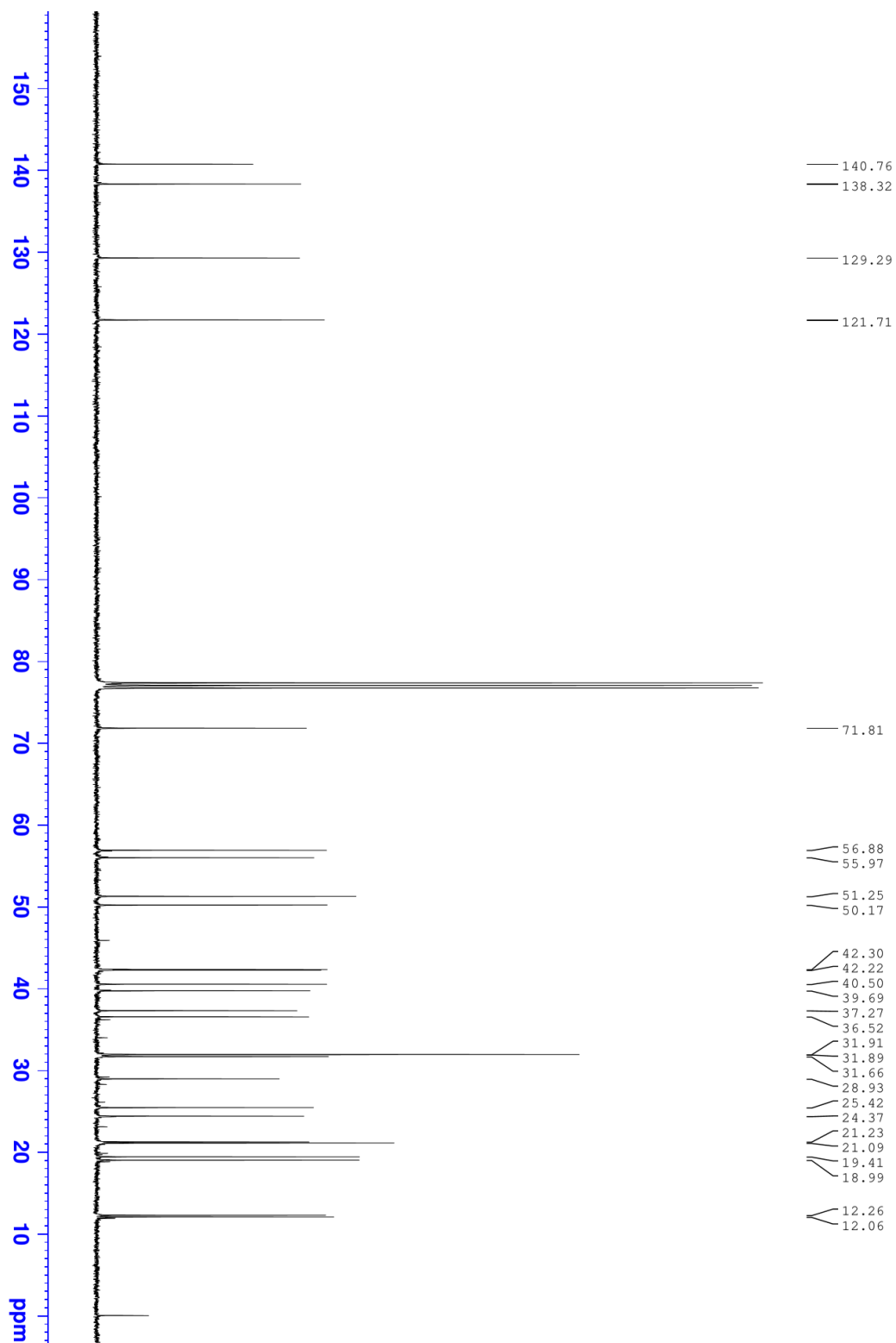

**S12 Figure:** <sup>13</sup>C NMR spectrum of compound (2) in CDCl<sub>3</sub> (400 MHz).

Supplement: S12 Fig — (PDF) [file pone.0163270.s012.pdf]

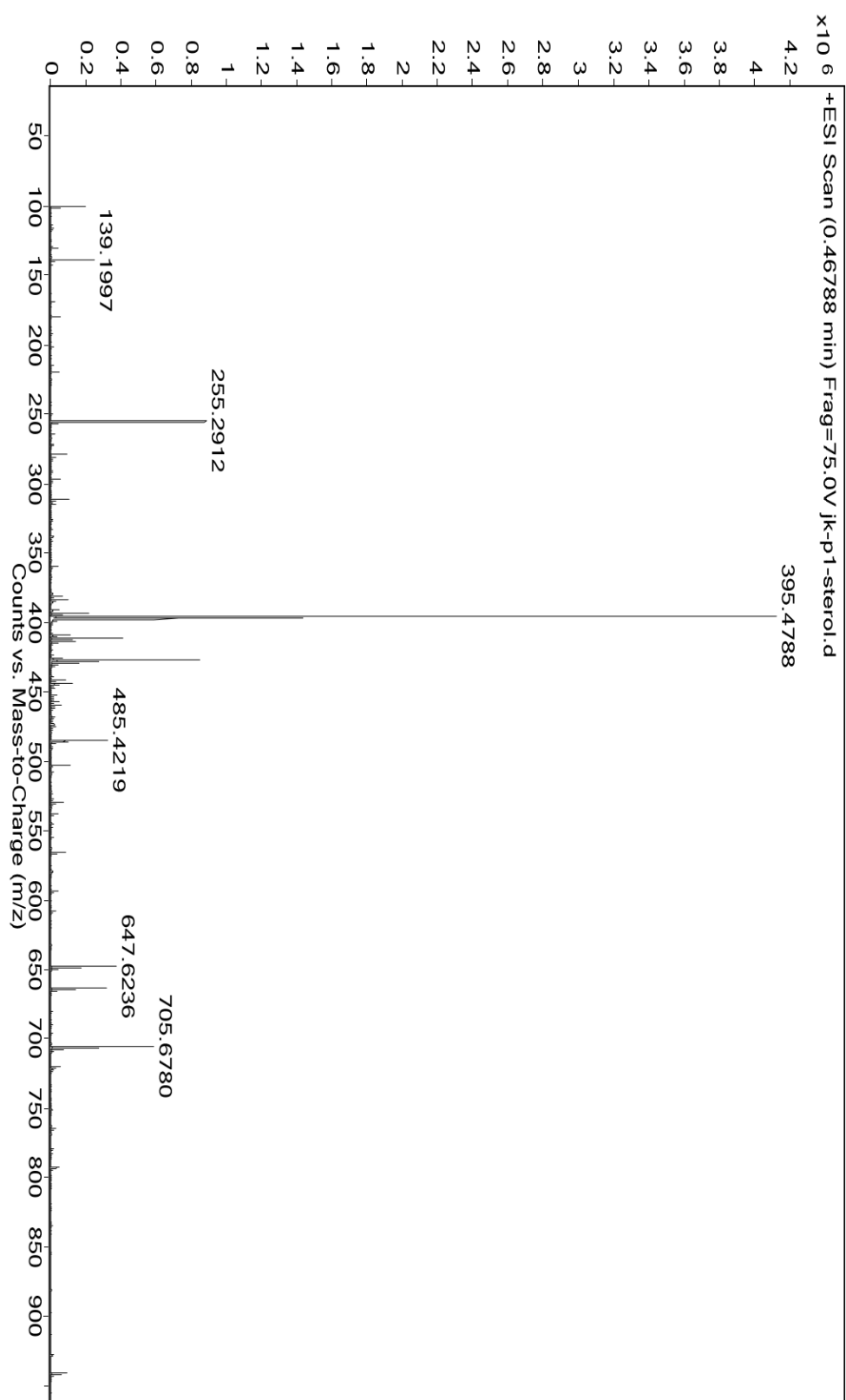

**S13 Figure:** HRMS (ESI-TOF) data for compound **2**.

Supplement: S13 Fig — (PDF) [file pone.0163270.s013.pdf]
