# Supplementary material for: Single Plant Derived Nanotechnology for Synergistic Antibacterial Therapies
Source: PLoS One. 2016 Sep 29;11(9):e0163270. doi: 10.1371/journal.pone.0163270 (PMC5042556; doi:10.1371/journal.pone.0163270)
Supplement: S11 Fig — (PDF) [file pone.0163270.s011.pdf]

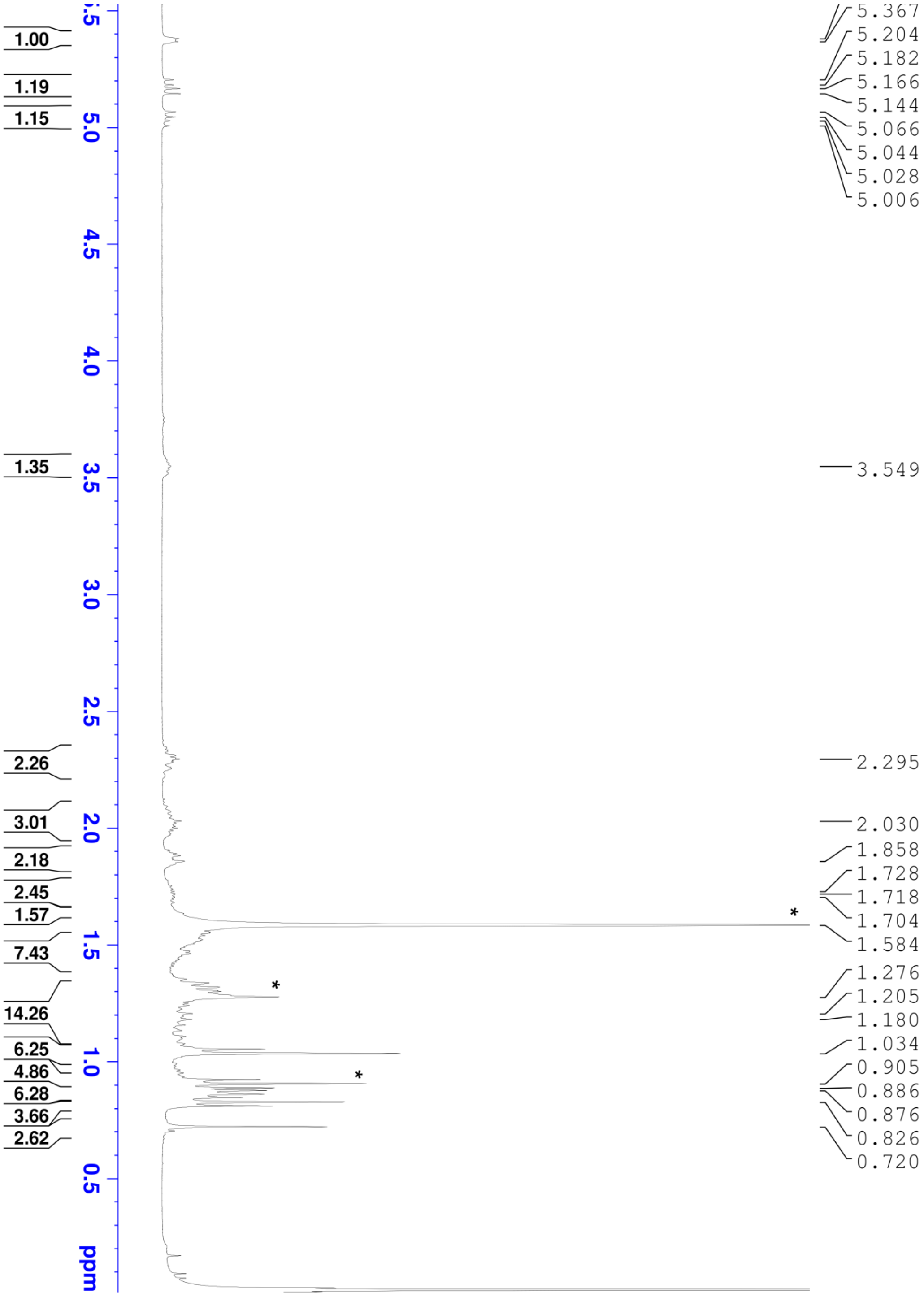

S11 Figure: ^{1}H NMR spectrum of compound **(2)** in $CDCl_3$ (400 MHz). Peaks noted with asterisks (*) indicate the presence of some residual solvent in the regions indicated.
